# Two new entomopathogenic species of *Ophiocordyceps* in Thailand

**DOI:** 10.3897/mycokeys.47.29898

**Published:** 2019-02-21

**Authors:** Yuan-Pin Xiao, Sinang Hongsanan, Kevin D. Hyde, Siraprapa Brooks, Ning Xie, Feng-Yao Long, Ting-Chi Wen

**Affiliations:** 1 Engineering Research Center of Southwest Bio-Pharmaceutical Resources, Ministry of Education, Guizhou University, Guiyang, Guizhou Province 550025, China; 2 Center of Excellence in Fungal Research, Mae Fah Luang University, Chiang Rai 57100, Thailand; 3 School of Science, Mae Fah Luang University, Chiang Rai 57100, Thailand; 4 Shenzhen Key Laboratory of Microbial Genetic Engineering, College of Life Sciences and Oceanography, Shenzhen University, Shenzhen 518060, China

**Keywords:** 2 new taxa, Hypocreales, morphology, phylogenetic, taxonomy

## Abstract

*Ophiocordyceps* is entomopathogenic and the largest studied genus in the family *Ophiocordycipitaceae*. Many species in this genus have been reported from Thailand. The first new species introduced in this paper, *Ophiocordycepsglobiceps*, differs from other species based on its smaller perithecia, shorter asci and secondary ascospores and additionally, in parasitising fly species. Phylogenetic analyses of combined LSU, SSU, ITS, TEF1α and RPB1 sequence data indicate that *O.globiceps* forms a distinct lineage within the genus *Ophiocordyceps* as a new species. The second new species, *Ophiocordycepssporangifera*, is distinguished from closely related species by infecting larvae of insects (Coleoptera, Elateridae) and by producing white to brown sporangia, longer secondary synnemata and shorter primary and secondary phialides. We introduce *O.sporangifera* based on its significant morphological differences from other similar species, even though phylogenetic distinction is not well-supported.

## Introduction

The genus *Ophiocordyceps* was introduced by Petch (1931) to accommodate species which have different features of asci and ascospores from *Cordyceps* (Petch 1931). *Ophiocordyceps* was treated as a subgenus of *Cordyceps* by Kobayasi (1941, 1982) and Mains (1958). Sung et al. (2007a) established the new family *Ophiocordycipitaceae* in Hypocreales (Sordariomycetes) and revised *Ophiocordyceps* as the type genus based on phylogenetic analyses. This is followed in the Outline of Ascomycetes (Wijayawardene et al. 2018). The main characters of the sexual morph species of *Ophiocordyceps* are fibrous, hard, pliant-to-wiry, dark stromata with superficial to immersed perithecia (Sung et al. 2007a, Ban et al. 2015). The asexual morphs in the majority of species have hirsutella-like and hymenostilbe-like features (Kepler et al. 2013, Maharachchikumbura et al. 2015, 2016). The hosts of species in *Ophiocordyceps* are larval lepidopterans and coleopterans, adult hymenopterans, hemipterans, dipterans, orthopterans or dragonflies (Odonata) and, in few cases, spiders (Kobayasi 1941, Mains 1958, Sung et al. 2007a, Ban et al. 2015). Hitherto, *Ophiocordyceps* included 233 species (Index Fungorum, June 2018) with a worldwide diversity (Sung et al. 2007a, Ban et al. 2015, Spatafora et al. 2015, Shrestha et al. 2017).

Thailand is located in the tropical areas with a rich biodiversity (Luangsa-ard et al. 2008, Aung et al. 2008, Luangsa-ard et al. 2010, Hyde et al. 2017, Hyde et al. 2018). A variety of entomopathogenic species (more than 400 species) (Index Fungorum, June 2018, Luangsa-ard et al. 2008, Luangsa-ard et al. 2010) were reported from Thailand after the first species recorded by Petch in 1932. In this study, we introduce two new species of *Ophiocordyceps*, which were found on larvae of insects (Lepidoptera, Cossidae) and adult Diptera. The descriptions of these two new species and phylogenetic evidence for the new taxa are provided. Morphological differences between two new species and their related species are also discussed.

## Methods

### Collection, isolation, and morphology study

Specimens were collected in The Mushroom Research Centre, Chiang Mai, Thailand, from soil and grass litter and taken to the laboratory. Fruiting bodies were examined using free hand sections under a stereomicroscope. Water-mounted slides were prepared for a microscope study and photographed under a compound microscope. Strains were isolated from single spores by using the protocol in Chomnunti et al. (2014). Cultures were incubated at 25 °C for 4–10 weeks on potato extract agar (PDA) in light-promoted sporulation.

### DNA extraction, PCR amplification and determination of DNA sequences

DNA was extracted from both dried specimens and cultures by using E.Z.N.A.TM Fungal DNA MiniKit (Omega Biotech, CA, USA), according to the manufacturers protocols. Universal known primers were used in PCR amplification; ITS4/ITS5 for internal transcribed spacer gene region (ITS), NS1/NS4 for partial small subunit ribosomal RNA gene region (SSU), LROR/LR5 for partial large subunit rDNA gene region (LSU) (Vilgalys and Hester 1990, White et al. 1990), 983F/2218R for partial translation elongation factor 1-alpha gene region (TEF1α) (Sung et al. 2007b) and CRPB1A/RPB1Cr for partial RNA polymerase II largest subunit gene region (RPB1) (Castlebury et al. 2004). PCR products were sequenced by Sangon Biotech (Shanghai) Co., Ltd., Shanghai, China. Specimen was performed by using TaKaRa PMD18-T vector system (TaKaRa Biotechnology, Dalian, China), while PCR products could not be sequenced directly.

### Phylogenetic analyses

Sequence data were obtained from GenBank based on previous studies as listed in Table 1. MAFFT v.7 was used to align combined datasets of ITS, SSU, LSU, TEF1α and RPB1 regions (Katoh and Standley 2013, http://mafft.cbrc.jp/alignment/server/). BioEdit (Hall 2011) was used to check alignment manually. Gaps were treated as missing data. *Tolypocladiuminflatum* W. Gams and *T.ophioglossoides* (J.F. Gmel.) C.A. Quandt et al. (Kepler et al. 2012, Schoch et al. 2012) were selected as outgroup taxa.

**Table 1.** Sources of isolates and GenBank accession numbers used in the paper.

Species	Insecta	Voucher	SSU	ITS	LSU	TEF1α	RPB1	References
* H. dipterigena *	Diptera	NHJ12170.02		GU723771		GU797126		Luangsa-ard et al. 2011
* O. acicularis *	Coleoptera (larva)	OSC 110988	EF468951		EF468804	EF468745	EF468853	Sung et al. 2007a
* O. agriotidis *	Coleoptera (larva)	ARSEF 5692	DQ522540	JN049819	DQ518754	DQ522322	DQ522368	Ban et al. 2015
* O. amazonica *	Orthoptera (Acrididae imago)	Ophama2026	KJ917562		KJ917571	KM411989	KP212902	Sanjuan et al. 2015
* O. annulata *	Coleoptera	CEM 303	KJ878915		KJ878881	KJ878962	KJ878995	Quandt et al. 2014
* O. aphodii *	Coleoptera	ARSEF 5498	DQ522541		DQ518755	DQ522323		Spatafora et al. 2007
* O. appendiculata *	Coleoptera (larva)	NBRC 106960	JN941728	JN943326	JN941413	AB968577	JN992462	Ban et al. 2015
* O. arborescens *	Cossida (larva)	NBRC 105891		AB968398	AB968414	AB968572		Ban et al. 2015
* O. australis *	Hymenoptera (ant)	Ophaus992	KC610785		KC610766	KC610731	KF658663	Ban et al. 2015
* O. barnesii *	Coleoptera (larva)	BCC28560	EU408776				EU408773	Luangsa-ard et al. 2010
* O. brunneinigra *	Hemiptera (Cicadellidae)	TBRC 8093			MF614654	MF614638	MF614668	Luangsa-Ard et al. 2018
* O. brunneiperitheciata *	Lepidoptera (larva)	TBRC 8100		MF614658		MF614643		Luangsa-Ard et al. 2018
* O. brunneipunctata *	Coleoptera (Elateridae larva)	OSC 128576	DQ522542		DQ518756	DQ522324	DQ522369	Spatafora et al. 2007
* O. buquetii *	Hymenoptera (Formicidae)	HMAS 199613	KJ878939		KJ878904	KJ878984	KJ879019	Quandt et al. 2014
* O. citrina *	Hemiptera	TNS F18537			KJ878903	KJ878983		Quandt et al. 2014
* O. clavata *	Coleoptera (larva)	NBRC 106962	JN941726	JN943328	JN941415	AB968587	JN992460	Schoch et al. 2012
* O. coccidiicola *	Insect	NBRC 100682	AB968404		AB968419	AB968583		Ban et al. 2015
* O. coccidiicola *	Insect	HMAS199612	KJ878917	AB027377	KJ878884	KJ878965	KJ878998	Quandt et al. 2014
* O. coenomyia *	Coenomyia (larva)	NBRC 108993	AB968384	AB968396	AB968412	AB968570		Ban et al. 2015
* O. communis *	Coleoptera	NHJ 12581	EF468973		EF468831	EF468775		Quandt et al. 2014
* O. cossidarum *	Lepidoptera (larva)	MFLU 17-0752	MF398186		MF398187	MF928403	MF928404	Hyde et al. 2017
* O. crinalis *	Lepidopteran (larva)	HIMGD17327		EU149926				Zhang et al. 2007
* O. curculionum *	Coleoptera (adult Curculionidae)	OSC 151910	KJ878918		KJ878885		KJ878999	Quandt et al. 2014
* O. cylindrospora *	Hymenoptera (adult wasp)	MFLU: 17-1961	MG553651	MG553635	MG553652			Hyde et al. 2018
* O. dipterigena *	Diptera (adult fly)	MY621		GU723764		GU797126		Luangsa-ard et al. 2011
* O. dipterigena *	Diptera (adult fly)	MRCIF71		EU573346				Freire 2015
* O. dipterigena *	Diptera (adult fly)	OSC 151912	KJ878920		KJ878887	KJ878967	KJ879001	Quandt et al. 2014
* O. elongata *	Lepidoptera (larva)	OSC 110989			EF468808	EF468748	EF468856	Sung et al. 2007a
* O. emeiensis *	Lepidoptera (larva)	G96031		AJ309347				Liu et al. 2002
* O. entomorrhiza *	Lepidoptera	KEW 53484	EF468954	JN049850	EF468809	EF468749	EF468857	Quandt et al. 2014
* O. evansii *	Hymenoptera (Pachycondylaharpax)	Ophsp 858	KC610796		KC610770	KC610736	KP212916	Sanjuan et al. 2015
* O. forquignonii *	Diptera (adult fly)	OSC 151908	KJ878922		KJ878889		KJ879003	Quandt et al. 2014
* O. formicarum *	*Camponotus* (Ant)	BCMU CF 01		AB222678				Freire 2015
* O. formicarum *	*Camponotus* (Ant)	BCMU CF 02		AB222679				Freire 2015
* O. formosana *	Coleoptera (larva)	MFLU: 15-3888						Li et al. 2016
* O. fulgoromorphila *	Hemiptera (Fulgoridae adult)	Ophara717	KC610794		KC610760	KC610729	KF658676	Sanjuan et al. 2015
* O. geometridicola *	Lepidoptera (Geometridae)	TBRC 8095			MF614648	MF614632	MF614663	Luangsa-Ard et al. 2018
* O. globiceps *	Diptera (adult fly)	MFLUCC 18-0495	MH725811	MH725815	MH725829	MH727387		This study
* O. globiceps *	Diptera (adult fly)	MFLU 18-0661	MH725812	NH725816	MH725830	MH727388		This study
* O. gracilis *	Lepidoptera (larva)	EFCC 8572	EF468956	JN049851	EF468811	EF468751	EF468859	Kepler et al. 2012
* O. hemisphaerica *	Diptera (adult fly)	FLOR 59525	KX197233					Hyde et al. 2016
* O. heteropoda *	Hemiptera (cicada nymph)	OSC 106404	AY489690		AY489722	AY489617	AY489651	Castlebury et al. 2004
* O. irangiensis *	Hymenoptera (adult ant)	OSC 128579	EF469123		EF469076	EF469060	EF469089	Sung et al. 2007a
* O. issidarum *	Hemiptera (adult)	MFLU:17-0751		MF398185	MF398188			Hyde et al. 2017
* O. karstii *	*Hepialus* (larva)	MFLU:15-3884	KU854952			KU854945	KU854943	Li et al. 2016
* O. konnoana *	Coleoptera (larva)	EFCC 7315	EF468959			EF468753	EF468861	Sung et al. 2007a
* O. lanpingensis *	*Hepialus* (larva)	YHOS0707	KC417459		KC417461	KC417463	KC417465	Chen et al. 2013
* O. lloydii *	Hymenoptera (Camponotus)	OSC 151913	KJ878924		KJ878891	KJ878970	KJ879004	Quandt et al. 2014
* O. longissima *	Hemiptera (cicada nymph)	NBRC 108989	AB968394	AB968407	AB968421	AB968585		Sanjuan et al. 2015
* O. macroacicularis *	lepidopterans (larvae)	NBRC 105888	AB968389	AB968401	AB968417	AB968575		Ban et al. 2015
* O. melolonthae *	Coleoptera (Scarabeidae larva)	OSC 110993	DQ522548		DQ518762	DQ522331	DQ522376	Spatafora et al. 2007
* O. multiperitheciata *	Lepidoptera (larva)	BCC 69008			MF614657	MF614641		Luangsa-Ard et al. 2018
* O. myrmecophila *	Hymenoptera (adult ant)	MFLU 16-2912	MF351730	MF351726	MF372585	MF372759		Xiao et al. 2017
* O. myrmicarum *	*Formicidae* (adult ant)	ARSEF11864	KJ680150			JX566973	KJ680151	Simmons et al. 2015
* O. neovolkiana *	Coleoptera	OSC 151903	KJ878930		KJ878896	KJ878976	KJ879010	Quandt et al. 2014
* O. nigra *	Hemiptera	TNS 16252	KJ878941		KJ878906	KJ878986		Quandt et al. 2014
* O. nigrella *	Lepidoptera (larva)	EFCC 9247	EF468963	JN049853	EF468818	EF468758	EF468866	Sung et al. 2007a
* O. nutans *	Hemiptera (Pentatomidae adult)	OSC 110994	DQ522549		DQ518763	DQ522333	DQ522378	Spatafora et al. 2007
* O. odonatae *	Odonata (Dragonfly)	TNS F18563	D86055	AB104725				Ito and Hirano 1997
* O. pauciovoperitheciata *	Lepidoptera (larva)	TBRC 8106			MF614652	MF614633		Luangsa-Ard et al. 2018
* O. pseudoacicularis *	Lepidoptera (larva)	TBRC 8102			MF614646	MF614630	MF614661	Luangsa-Ard et al. 2018
* O. pulvinata *	Hymenoptera (adult ant)	TNS-F 30044	GU904208			GU904209	GU904210	Quandt et al. 2014
* O. purpureostromata *	Coleoptera	TNS F18430	KJ878931		KJ878897	KJ878977	KJ879011	Quandt et al. 2014
* O. pseudolloydii *	Formicidae (adult ant)	MFLU 15-1425		MF351725		MF372758	MF372761	Xiao et al. 2017
* O. ramosissimum *	Lepidoptera (larva)	GZUHHN8	KJ028012	KJ028007		KJ028014	KJ028017	Wen et al. 2014
* O. ravenelii *	Coleoptera (larva)	OSC 110995	DQ522550		DQ518764	DQ522334	DQ522379	Spatafora et al. 2007
* O. rhizoidea *	Isoptera (adult termite)	NHJ 12529	EF468969		EF468824	EF468765	EF468872	Sung et al. 2007a
* O. robertsii *	Lepidoptera (Hepialidae larva)	KEW 27083			EF468826	EF468766		Sung et al. 2007a
* O. rubiginosiperitheciata *	Coleoptera (larva)	NBRC 106966	JN941704	JN943344	JN941437	AB968582	JN992438	Ban et al. 2015
* O. sinensis *	Lepidopteran pupa	EFCC7287	EF468971	JN049854		F468767	EF468874	Sung et al. 2007a
* O. sobolifera *	Hemiptera (cicada nymph)	NBRC 106967	AB968395	AB968409	AB968422	AB968590		Ban et al. 2015
*O. sp*		FMF147		KX197238				Freire 2015
*O. sp*		OSC 110997	EF468976			EF468774	EF468879	Quandt et al. 2014
* O. spataforae *	Hemiptera (Fulgoridae)	NHJ 12525	EF469125		EF469078	EF469063	EF469092	Sung et al. 2007a
* O. sphecocephala *	Hymenoptera (adult wasp)	NBRC 101753	JN941695	JN943350	JN941446	AB968592	JN992429	Ban et al. 2015
* O. sporangifera *	Lepidoptera (Cossidae)	MFLUCC 18-0492	MH725814	MH725818	MH725832	MH727390	MH727392	This study
* O. sporangifera *	Lepidoptera (Cossidae)	MFLU 18-0658	MH725813	MH725817	MH725831	MH727389	MH727391	This study
* O. stylophora *	Coleoptera (Elateridae larva)	OSC 111000	DQ522552	JN049828	DQ518766	DQ522337	DQ522382	Spatafora et al. 2007
* O. superficialis *	Insect	MICH 36253	EF468983				EF468883	Sung et al. 2007a
* O. thanathonensis *	Hymenotera (adult ant)	MFU 16-29010	MF882926	MF850375	MF850375	MF872614	MF872616	Xiao et al. 2017
* O. tricentri *	Hemiptera (Cercopidae)	NBRC 106968	AB968393	AB968410	AB968423	AB968593		Ban et al. 2015
* O. unilateralis *	Hymenoptera (Camponotus)	OSC 128574	DQ522554		DQ518768	DQ522339	DQ522385	Spatafora et al. 2007
* O. variabilis *	Diptera (larva)	OSC 111003	EF468985		EF468839	EF468779	EF468885	Sung et al. 2007a
* O. xuefengensis *	Lepidoptera (Hepialidae larva)	GZUH2012HN19	KC631788	KC631803		KC631794	KC631799	Wen et al. 2013
* O. yakusimensis *	Hemiptera (cicada nymph)	HMAS 199604	KJ878938		KJ878902		KJ879018	Quandt et al. 2014
* T. inflatum *	Coleoptera (larva)	OSC 71235	EF469124	JN049844	EF469077	EF469061	EF469090	Kepler et al. 2012
* T. ophioglossoides *	Fungi (*Elaphomyces* sp.)	NBRC 106332	JN941732	JN943322	JN941409		JN992466	Schoch et al. 2012

**Figure 1.**
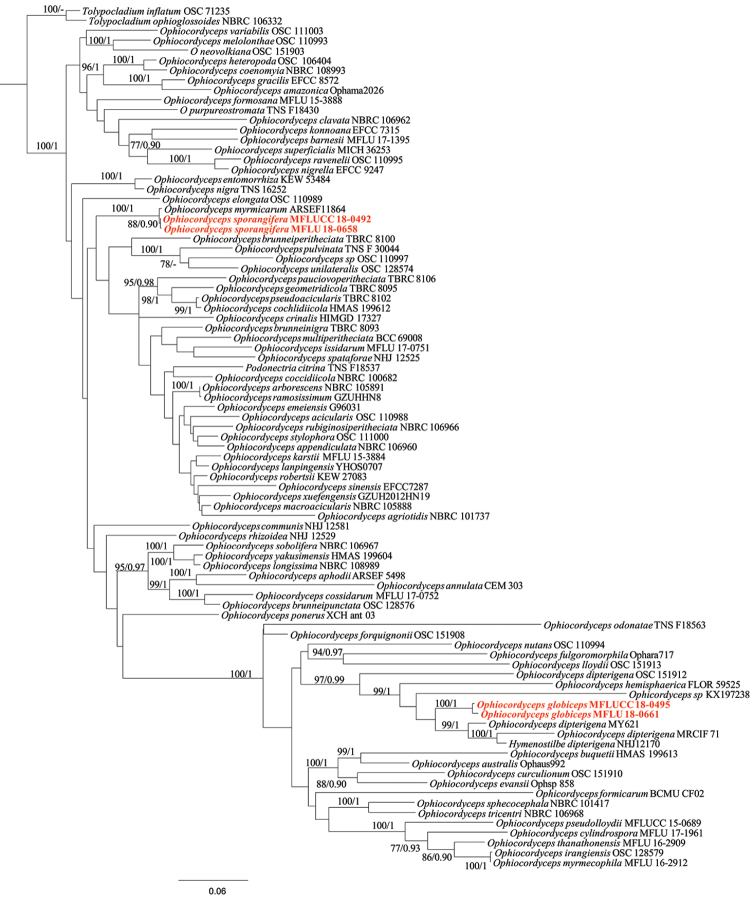
Phylogram of *Ophiocordycepsglobiceps* and *O.sporangifera* generated from maximum likelihood (RAxML) analysis of ITS, SSU, LSU, RPB1 and TEF1α sequence data. *Tolypocladiuminflatum* and *T.ophioglossoides* were used as outgroup taxon. Maximum likelihood bootstrap values greater than 75% and Bayesian posterior probabilities over 0.90 were indicated above the nodes. The new species are indicated in red.

Maximum likelihood trees (ML) were estimated by using the software RAxML 7.2.8 Black Box (Stamatakis 2006, Stamatakis et al. 2008) in the CIPRES Science Gateway platform (Miller et al. 2010). MrModeltest v.2.3 (Nylander 2004) was used to determine the best-fit model of evolution for Bayesian analyses. MrBayes v.3.1.2 (Ronquist and Huelsenbeck 2003) was used to evaluate posterior probabilities (PP) (Rannala and Yang 1996, Zhaxybayeva and Gogarten 2002) by Markov Chain Monte Carlo sampling (BMCMC). Six simultaneous Markov chains were run for 10,000,000 generations, trees were sampled every 100^th^ generation and 100,001 trees were obtained. The first 25% of trees (25,000) were discarded, as they represented the burn-in phase of the analyses, while the remaining trees (75,001) were used for calculation of posterior probabilities in the majority rule consensus tree (critical values for the topological convergence diagnostic is 0.01). Trees were figured in FigTree v1.4.0 programme (Rambaut 2012). Bayesian Posterior Probabilities (BYPP) equal to or great than 0.90 were given below each node (Fig. 1).

## Results

### Molecular phylogeny

Eighty-seven taxa (including the four with new sequence data) were included in the combined ITS, SSU, LSU, RPB1 and TEF1α dataset (Table 1), which comprises 3894 characters with gaps; 1011 characters for SSU, 824 for LSU, 561 for ITS, 880 for TEF1α and 618 for RPB1. Tree topology of the RAxML analysis was similar to the Bayesian analysis. The best scoring RAxML tree with a final likelihood value of -46932.268101 is presented (Fig. 1). The matrix had 2081 distinct alignment patterns, with 35.22% of undetermined characters or gaps. Parameters for the GTR model of the concatenated dataset were as follows: Estimated base frequencies; A = 0.240006, C = 0.270755, G = 0.276725, T = 0.212514; substitution rates AC = 1.073676, AG = 3.611556, AT = 1.170890, CG = 1.176549, CT = 6.339087, GT = 1.000; gamma distribution shape parameter α = 0.265589.

### Taxonomy

#### Ophiocordyceps globiceps

Taxon classificationFungiHypocrealesOphiocordycipitaceae

Y.P. Xiao, T.C. Wen & K.D. Hyde sp. nov.

Index Fungorum number: IF555323

Faces of fungi number: FoF 04864

Fig. 2

##### Etymology.

The specific epithet refers to the feature of the secondary hemispherical to globoid fertile head.

**Sexual morph**: *Stromata* 4–8 mm long × 0.5–1 mm diam., one or several from the host, stipitate, capitate, unbranched, cinnamon to yellow. *Stipe* 3.5–7.5 mm long, 0.2–0.5 mm diam., yellow, cylindrical, with a fertile apex. *Fertile head* 1–1.5 mm long, 1–1.2 mm diam., cinnamon to yellow, single, hemispherical to globoid. *Perithecia* 538–663 × 182–247 μm (x̄= 600 × 214 µm, n = 60), immersed, ovoid to elongated pyriform, thick-walled, vertical with the ostioles opening on the upper surface of the head. *Peridium* 17–22 µm (x̄ = 20 µm, n = 90) wide, hyaline, of *textura porrecta* to *textura prismatica* to *textura angularis*. *Asci* 373–454 × 5.7–8.2 μm (x̄ = 413 × 7 µm, n = 90), 8-spored, hyaline, filiform, with a thick apex. *Apical cap* 4.4–6.4 × 4.9–5.7 μm (x̄ = 5.4 × 5.3 µm, n = 60), thick, with a small channel in the centre. *Ascospores* 240–303 × 1.8–2.3 μm (x̄ = 272 × 2.1 µm, n = 60), filiform, hyaline, multiseptate. *Secondary ascospores* 4–5.4 × 1.2–1.9 μm (x̄ = 4.7 × 1.6 µm, n = 90) cylindrical to fusoid, 1-celled, straight, hyaline, smooth-walled. **Asexual morph**: Undetermined.

**Figure 2.**
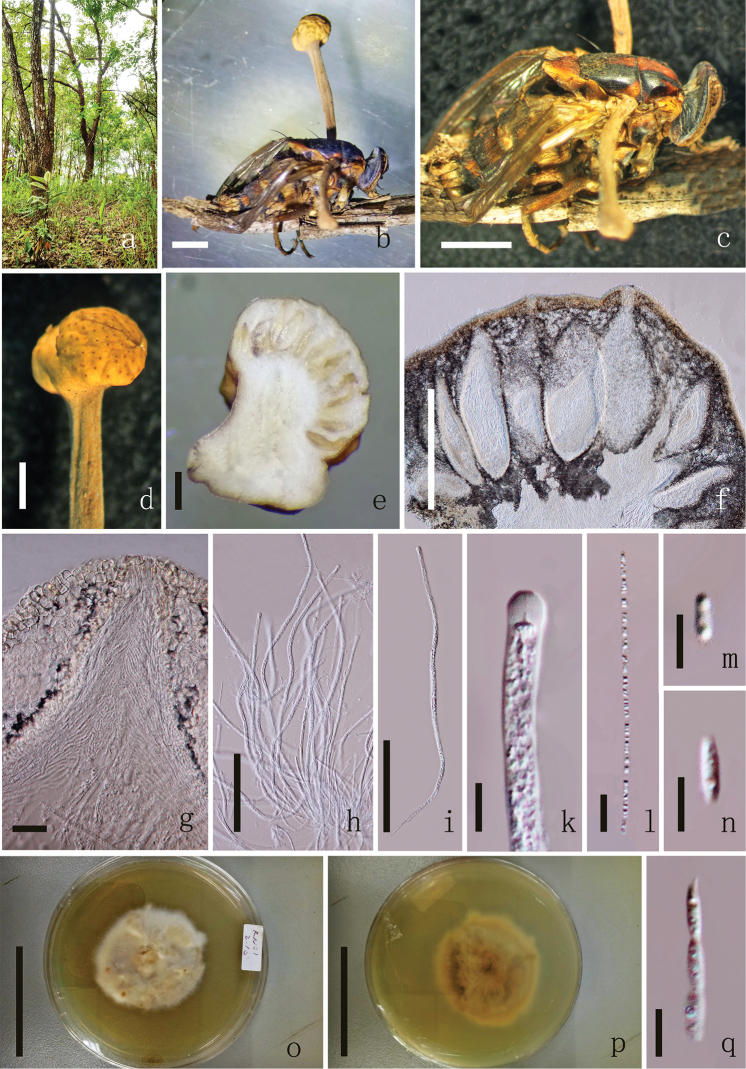
*Ophiocordycepsglobiceps* (holotype MFLU 18–0661). **a** Habitat **b** Ascostroma emerging from infected fly **c** Host **d** Fertile head of ascostroma **e** Vertical section of the stroma **f** Section of ascomata **g** Peridium **h, i** Asci **k** Apical cap of asci **l, q** Part of ascospore **m, n** Secondary ascospores **o** Upper side of the culture **p** Reverse side of the culture. Scale bars: 1000 µm (**b–d**), 500 µm (**e, f**), 100 µm (**h, i**), 20 µm (**g**), 10 µm (**k, l**), 5 µm (**m, n, q**), 5 cm (**o, p**).

##### Culture characteristics.

growing on PDA, reaching 5 cm diam., after 6 weeks at 25 °C, superficial cottony, whitened, loose, reverse yellow. After 10 weeks at 25 °C, reaching 6 cm diam., no conidiogenous structures observed.

##### Material examined.

THAILAND, Ranong, Tambon Khao Niwet, parasitise on fly (Muscidae, Diptera) 7 mm long, 3 mm wide, brown to dark brown, without hyphae on the surface, collected on the grass stem, 19 July 2015, YuanPin Xiao, (MFLU 18–0661, **holotype**, ex-type living culture, MFLUCC 18–0495); Chiang Mai, Thailand, on adult fly (Diptera), 6.5 mm long, 2.7 mm wide, brown to dark brown, without hyphae on the surface, collected on the grass, 19 July 2017, YuanPin Xiao, (MFLU 18–0662, **paratypes**, living culture MFLUCC 18–0496).

##### Notes.

In the phylogenetic tree, *Ophiocordycepsglobiceps* is closely related to *O.dipterigena* (Berk. & Broome) G.H. Sung, J.M. Sung, Hywel-Jones & Spatafor. (Thailand) and *O.hemisphaerica* Mafalda-Freire, Reck & Drechsler-Santos (Brazil), which infect flies (Luangsa-ard et al. 2008, Hyde et al. 2016). *Ophiocordycepsglobiceps* also groups with *Ophiocordyceps* sp. (FMF147) (106bp ITS differ), which was introduced by ITS sequence data and without any other detail (Freire 2015). *Ophiocordycepsglobiceps* has 60 bp that differ from *O.dipterigena* (MY621, Thailand) in the ITS region, 19 bp in TEF1α. It has 87 bp that differ from *Hymenostilbedipterigena* Petch (NHJ12170, Thailand, asexual morph of *O.dipterigena*) in the ITS region and 20 bp in TEF1α. *Ophiocordycepsglobiceps* also has 94 bp (ITS) that differ from *O.dipterigena* (MRCIF71, Thailand), which only has ITS and without any details. *Ophiocordycepsglobiceps* has 104 bp that differ from *O.hemisphaerica* (FLOR 59525) in the ITS region and has 21 bp in nr*SSU*, 97 bp in nr*LSU*, 74 bp in TEF1α that differ from *O.dipterigena* (OSC 151913).

We compared the new species with other *Ophiocordyceps* species which infect flies (Diptera) or are morphologically similar to *O.globiceps* (Table 2). *Ophiocordycepsglobiceps* differs from three records of *O.dipterigena* found in Sri Lanka, Japan and Thailand by producing single smaller stroma, smaller and shorter perithecia, shorter asci and smaller ascospores (Table 2). *Cordycepssakishimensis* Kobayasi & Shimizu, *Ophiocordycepsdiscoideicapitata* (Kobayasi & Shimizu) G.H. Sung, J.M. Sung, Hywel-Jones & Spatafora, *Ophiocordycepsforquignonii* (Quél.) G.H. Sung, J.M. Sung, Hywel-Jones & Spatafora, *Ophiocordycepshemisphaerica* Mafalda-Freire, Reck & Drechsler-Santos, *Ophiocordycepslacrimoidis* Mafalda-Freire, Reck & Drechsler-Santos and *Cordycepsmuscicola* Möller (= *Ophiocordycepsmuscicola*) have been reported as fly infected taxa (Saccardo 1891, Möller 1901, Kobayasi and Shimizu 1982, Freire 2015, Hyde et al. 2016), but their morphology is different from *O.globiceps* (see Table 2). *Cordycepssakishimensis* is distinct from *O.globiceps* in having white, longer, cylindrical stromata and larger superficial perithecia. *Ophiocordycepsdiscoideicapitata* differs from *O.globiceps* by producing smaller stromata, pyriform, larger perithecia and longer part-spores (Table 2) (Kobayasi and Shimizu 1982). *Ophiocordycepsforquignonii* is distinct from *O.globiceps* in having a cylindrical fertile apex and oval secondary ascospores (Table 2) (Saccardo 1891). Molecular data indicate that the new species has 26 bp in nrSSU and 89 bp in nrLSU that are different from *O.forquignonii*. *Ophiocordycepshemisphaerica* is different from *O.globiceps* in having longer stomata, larger obpyriform perithecia, longer asci and longer fusoid part-spores (Hyde et al. 2016). *Ophiocordycepslacrimoidis* (Diptera infected species) was not considered in our phylogenetic sampling as the DNA (ITS) sequence did not align well with other species, but its DNA sequence differed by 154 bp in the ITS region from the sequence of *O.globiceps*. However, *Ophiocordycepslacrimoidis* is morphologically different from our new species in producing longer stipe, obpyriform, slightly curved perithecia, longer asci and longer part spores. *Cordycepsmuscicola* was revised as *Ophiocordycepsmuscicola* by Freire (2015), while it is different from *O.globiceps* in having longer stromata, larger pyriform perithecia, longer asci and longer part-spores (Möller 1901, Freire 2015). We would like to introduce *Ophiocordycepsglobiceps* as a new species based on the phylogenetic and morphological analyses.

**Table 2.** Synopsis of *Ophiocordyceps* species discussed in the paper.

Species	Location	Host	Stromata (mm)	Stipe (mm)	Fertile part (mm)	Perithecia (μm)	Asci (μm)	Ascospores (μm)	Part-spores (μm)	Reference
* C. sakishimensis *	Japan	Diptera	6–7 long, cylindrical, white			500 × 250–260, superficial, ovoid			4–6 × 1, cylindrical	Kobayasi and Shimizu 1983
*O.dipterigena* (First record)	Sri Lanka		5–10 × 1, pale	Cylindrical	Globose				10 × 1.5	Berkeley and Broome 1873, Freire 2015
* O. dipterigena *	Japan	Diptera	5–8 long, 1–2 wide, 0.5–1 wide, orange-cinnamon or cinnamon-brown	0.2–0.5 thick, orange-cinnamon to light yellow		Narrowly ovoid or conoid, 700–900 × 240–400, wall 15–25 thick	480–600 long	Filiform, multiseptate	6–12 × 1–1.5, cylindric or fusoid fragments	Kobayasi 1941
* O. dipterigena *	Thailand	Diptera	4–10 long, pale cream-yellow to orange-brown		1–1.5 high, 1.5–2.5 diam., terminal, disc-like to subglobose	800–1000 × 200–300, narrowly ovoid to obclavate	450–600 × 4–6, cylindrical	Filiform, breaking up into 64 part-spore	6–12 × 1–1.5, cylindrical to fusiform	Luangsa-ard et al. 2008
* O. discoideicapitata *	Japan	Diptera	2.5–3.5 × 0.7–1.2, two		3–4, discoid, laterally conical	620–700 × 200–250, pyriform	5–6 diam., filiform		6–9 × 1, cylindrical, truncated	Kobayasi and Shimizu 1982
* O. forquignonii *		Diptera		3-6 long, subfiliform, with a cylindrical apex	Cylindrical	Ellipsoid			Oval, 8	Saccardo 1891
* O. globiceps *	Thailand	Diptera	4–8 long × 0.5–1 diam., unbranched, cinnamon to yellow, one or several from host	3.5–7.5 long, 0.2–0.5 diam., cinnamon to yellow, cylindrical, with a fertile apex	1–1.5 long, 1–1.2 diam., yellow, hemispherical to globoid	538–663 × 182–247, ovoid to elongated pyriform	373–454 × 5.7–8	240–303 × 1.8–2.3, filiform, hyaline, multiseptate	4–5.4 × 1.2–1.9, cylindrical to fusoid	This study
* O. hemisphaerica *	Brazil	Diptera (Muscidae)	12–20 × 0.8–1, unbranched, brown to greyish-brown	11–19 long, 0.8–1 wide, cylindrical, with a fertile apex	1–1.2 long, 2–4 diam., hemispherical	780–860 × 220–290, Obpyriform, slightly curved	500–640 × 5–6	Filiform, more than 52 septa	7–10 × 1–1.5, cylindrical to unusually fusoid	Hyde et al. 2016
* O. lacrimoidis *	Brazil	Diptera	4–5 × 1, two, simple	3–4 long, 1 wide, cylindrical, epidermal layer brown, medullar region white to cream	1.2 long, 1.8–2.2 diam., discoid, pale to dark yellowish	650–700 × 200–250, immersed, obpyriform, slightly curved	350–450 × 5, narrow cylindrical	Filiform, as long as asci, hyaline, more than 56 septa	8–14 × 2, cylindrical, hyaline	Hyde et al. 2016
*O.muscicola* = *C.muscicola*	Brazil	Diptera	9–13 × 0.5–1, two to six, rarely branched		2–4 × 1–1.2, discoid	850–920 × 230–300, pyriform	550–700 × 5, filiform	650–700 × 2, 64 part-spores	11–14 × 2, terminal cylindrical, intermediates fusoids 8–10 × 1–2	Möller 1901, Freire 2015

#### Ophiocordyceps sporangifera

Taxon classificationFungiHypocrealesOphiocordycipitaceae

Y.P. Xiao, T.C. Wen & K.D. Hyde sp. nov.

Index Fungorum number: IF555324

Faces of fungi number: FoF 04865

Figs 3 , 4

##### Etymology.

The specific epithet refers to the feature of the sporangium-bearing.

**Sexual morph**: Unknown. **Asexual morph**: *Primary synnema* 9–18 cm high 1–2 mm diam., arising from the head region of the larva, branching into 2–5, cylindrical, brown to deep brown, with small white fertile head on the top, not smooth. *Fertile head* 500–2000 µm long, 400–1000 µm diam., globose to subglobose, capitulum, white to brown, arising from the apical end of primary synnema, mess of sporangium on the surface. *Sporangium* 78–121 µm diam. (x̄ = 100 µm, n = 60), spherical, arising from the apical end of primary synnema, white colour when immature, becoming brown to dark brown after maturity, consisting of thick-walled cells. *Secondary synnemata* 1092–1937 × 21–34 µm, (x ‒= 1515 × 27 µm, n = 60), laterally from the primary synnema, brown to white, cylindrical, not smooth. *Hyphae* 1.8–2.8 µm wide (x̄ = 2.3 µm, n = 60), irregularly multi-septate, brown, cylindrical, smooth or rough, sometimes particularly expand. *Phialides* 25–40 × 1.3–2.5 µm (x̄ = 33 × 1.9 µm, n = 60), hirsutella-like, hyaline, solitary, unbranched, narrow slender, smooth. *Conidia* 6.7–9.8 × 2.5–3.8 µm (x̄ = 8.3 × 3.2 µm, n = 60), 1 cell, hyaline, subglobose to reniform, bound in mucilaginous spheres. *Mucilaginous spheres* 10.5–12.9 × 6.4–8.7 µm (x̄ = 11.7 × 7.5 µm, n = 60), composed of 1–12 conidia, hyaline, at phialide apex.

**Figure 3.**
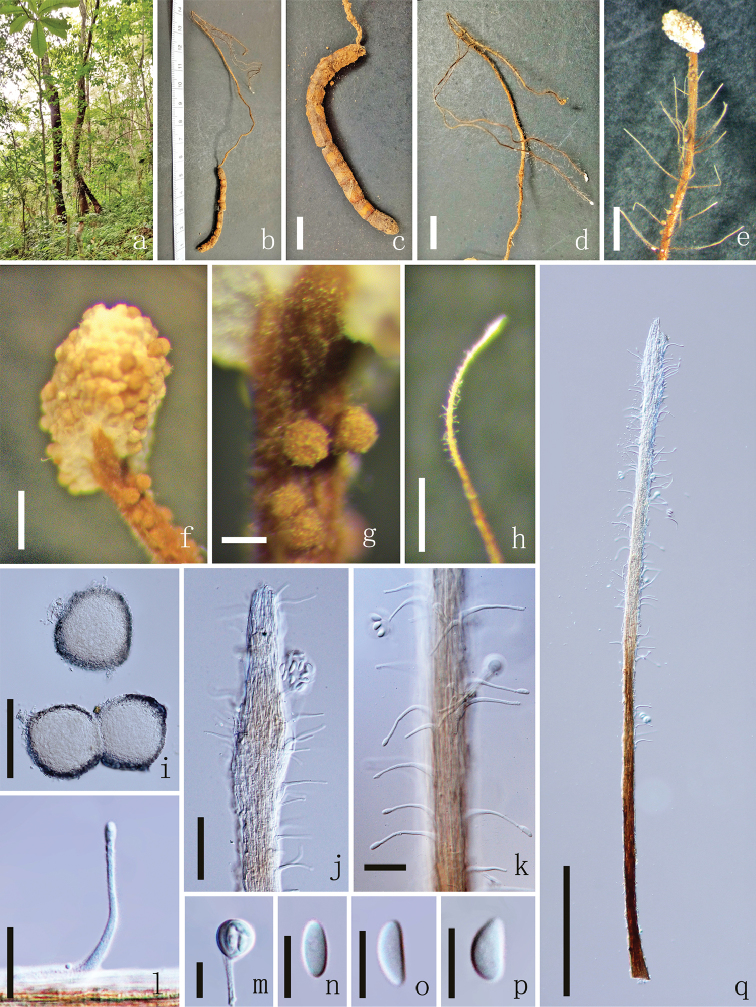
*Ophiocordycepssporangifera* (holotype MFLU 18–0658). **a** Habitat **b** Synnemata on host surface **c** Host **d, e** Synnemata **f** Fertile head of primary synnema **g** Sporangium **h** Secondary synnema **i** Sporangium **j, k, q** Part of secondary synnema **l** Phialides **m** Conidia bound by deliquescing mucilaginous material **n–p** Conidia. Scale bars: 1 cm (**c, d**), 1000 µm (**e**), 200 µm (**f, h, q**), 100 µm (**g, i**), 50 µm (**j**), 20 µm (**k, l**), 10 µm (**m–p**).

##### Culture Characteristics.

growing on PDA, reaching 2 cm diam., after 4 weeks at 25 °C,with circular, dense mycelium on the surface. After 6 weeks, the colour of the colony gradually deepened from white to dark brown from the periphery to the centre, with complex fold as 4 circle rings, reverse white to yellow in colour, with ring. Synnemata was produced after 8 weeks. Most of the characters are the same as the fresh collection except phialides and mucilaginous spheres. *Phialides* 56–86 µm long (x̄ = 71 µm, n = 60), 3–5 µm wide at base (x̄ = 4 µm, n = 60), 1.4–2.2 µm at top (x̄ = 1.8 µm, n = 60), hirsutella-like, hyaline, solitary, unbranched, narrow slender, smooth, 1–4 septa, not observed on host. *Mucilaginous spheres* 10.5–15.9 × 8.2–14.7 µm (x̄ = 12.7 × 11.5 µm, n = 60), 1–4 conidia, hyaline to brown. Observation stopped after 10 weeks.

##### Material examined.

THAILAND, Chiang Mai, The Mushroom Research Centre, on dead larva of Elateridae, Coleoptera, 6.5 cm long 0.38 cm diam., brown to dark brown, with thallus inside (larva), 18 July 2015, YuanPin Xiao, (MFLU 18–0658, **holotype**); THAILAND, Chiang Mai, The Mushroom Research Centre, on dead larva of Elateridae, Coleoptera, 5.8 cm long 0.4 cm diam., brown to dark brown, with thallus inside (larva), 22 August 2015, YuanPin Xiao, (MFLU 18–0659, **paratypes**, ex-type living culture, MFLUCC 18–0492); THAILAND, Chiang Mai, Samoeng on larva insect of Elateridae, Coleoptera, 5.5 cm long 0.32 cm diam., brown to dark brown, with thallus inside (larva), 18 June 2017, YuanPin Xiao, (MFLU 18–0660, **paratypes**, living culture, MFLUCC 18–0493, MFLUCC 18–0494).

##### Notes.

*Ophiocordycepssporangifera* is closely related to *O.myrmicarum* D.R. Simmons & Groden in our phylogenetic tree (Fig. 1). The morphology of *O.sporangifera* is different from *O.myrmicarum* in having longer primary and secondary synnemata, a white to brown sporangium, shorter phialides and it infects insect larvae (Lepidoptera, Cossidae), while *O.myrmicarum* was found on an ant (*Myrmicarubra*) (Simmons et al. 2015). The phylogenetic analysis does not have good support, but *O.sporangifera* is distinct from *O.myrmicarum*. In the phylogenetic tree, the relationships of *O.sporangifera* and *O.myrmicarum* are obscure because they share one clade with short branch length (100% ML/ 1 BYPP), while the two strains of *O.sporangifera* clustered together with a low bootstrap support (88% ML/ 0.90 BYPP). The type strain of *O.sporangifera* has 0 bp in nr*SSU*, 3 bp in TEF1α and 5 bp in RPB1 that are different from *O.myrmicarum*. However, the morphological features of those two species are different, thus, they should be treated as two separate species (Table 3).

**Figure 4.**
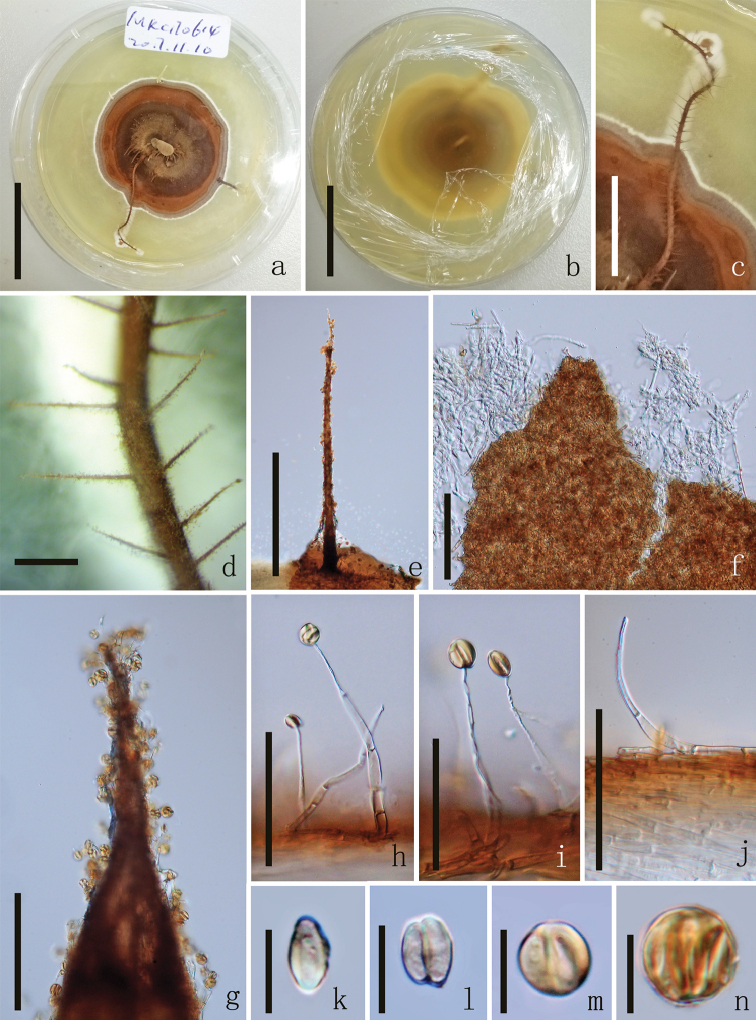
*Ophiocordycepssporangifera* (culture) MFLUCC 18–0492. **a** Upper side of the culture **b** Reverse side of the culture **c, d** Synnemata growing on PDA medium **e, g** Synnemata **f** Mycelium **h–j** Phialides **k** Conidia **l–n** Conidia form mucilaginous spheres. Scale bars: 1 cm (**a, b**), 5000 µm (**c**), 1000 µm (**d**), 500 µm (**e**), 100 µm (**f, g**), 50 µm (**h–j**), 10 µm (**k–n**).

**Table 3.** Synopsis of *Ophiocordyceps* species discussed in the paper.

Species	* Ophiocordyceps myrmicarum *	* Ophiocordyceps sporangifera *
Host	*Myrmicarubra* (Hymenoptera)	Elateridae, Coleoptera
Primary synnemata	Whitish-yellow aging to rufous brown	9–18 cm high 1–2 mm diam., brown to deep brown
Secondary synnemata (µm)	Hyaline aging to rufous brown, up to 350 long, narrow (25) at base, common on agar but not observed on host	Brown to white, not smooth 1092–1937 × 21–34, arising from the all parts of the primary synnemata, observed on both of the host and agar
Primary phialides (µm)	Subulate, hyaline or pigmented at base, 39.9–86.2 long, 3.6–5.4 wide at base	Slender, solitary, hyaline, unbranched, narrow, smooth, 25–40 × 1.3–2.5
Secondary phialides (µm)	Subulate, 27.2–47.0 long, 2.4–3.3 wide at base	Narrow slender, 56–86 long, 3–5 wide at base, 1.4–2.2 at top, 1–4 septa, common on culture but not observed on host
Sporangium (µm)	No observed	78–121 diam., spherical, white immature, brown after mature
Conidia (µm)	7.3–9.6 × 3.2–5.1 reniform to ovoid, bi-guttulate, aseptate	6.7–9.8 × 2.5–3.8, subglobose to reniform
Mucilaginous spheres (µm)	Composed of 1–4 conidia, hyaline to brown, at phialide apex	10.5–12.9 × 6.4–8.7, composed of 1–12 conidia, hyaline on host, 1–4 conidia on culture, hyaline to brown on culture
Reference	Simmons et al. 2015	This study

## Discussion

We introduce two new entomopathogenic species of *Ophiocordyceps*, one from Coleoptera (Elateridae) and the other from flies (Diptera). Morphological and phylogenetic analyses have provided insights to resolve generic delimitation (Sung et al. 2007a, Jeewon and Hyde 2016). Most of the species of this genus are parasitic on insects (Sung et al. 2007a, Maharachchikumbura et al. 2015, Wijayawardene et al. 2017). The sexual morph species in this genus is characterised by fibrous, hard, pliant-to-wiry, dark-coloured stroma with superficial to immersed perithecia (Sung et al. 2007a, Ban et al. 2015, Maharachchikumbura et al. 2015), while the asexual morph species have mainly hymenostilbe-like and hirsutella-like features, branched or unbranched phialides with oval to fusiform conidia (Kepler et al. 2013, Maharachchikumbura et al. 2015, 2016).

*Ophiocordycepsglobiceps* groups with *H.dipterigena*, *O.dipterigena*, *Ophiocordyceps* sp. and *O.hemisphaerica* in the phylogenetic tree with high bootstrap support, while four of these species are reported as fly (Diptera) parasitic fungi (Kobayasi 1941, Saccardo 1891, Luangsa-ard et al. 2011, Hyde et al. 2016). *Ophiocordycepsglobiceps* differs from closely related species by producing capitate, stipitate ascostromata, vertical, narrowly ovoid to obclavate, occasionally irregular perithecia and cylindrical secondary ascospores. Both morphology and phylogenetic analyses clearly show *O.globiceps* as a new species within *Ophiocordyceps*.

*Ophiocordycepssporangifera* is an asexual morph species and groups with *O.myrmicarum* in the phylogenetic tree (Fig. 1). *Ophiocordycepssporangifera* can be distinguished from *O.myrmicarum* by infecting and parasitising larvae of insects (Lepidoptera, Cossidae), producing white to brown sporangium, longer primary and secondary synnemata and shorter primary and secondary phialides. The new species can be defined based on the distinctive morphological characters even through the phylogenies are not well-supported (Jeewon and Hyde 2016). In case of intricate differences between a gene tree and a species tree and, in addition, several morphs can be under the influence of many genes which are not really being reflected in the phylogeny (Jeewon and Hyde 2016). In our study, morphological characters strongly support *O.sporangifera* as a new species within *Ophiocordyceps*, even through phylogenetic analysis is not well-resolved. In this case, other loci which have more phylogenetic variation than the current loci may be able to differentiate these two species.

## Supplementary Material

XML Treatment for Ophiocordyceps globiceps

XML Treatment for Ophiocordyceps sporangifera
